# Latent phenotypes pervade gene regulatory circuits

**DOI:** 10.1186/1752-0509-8-64

**Published:** 2014-05-30

**Authors:** Joshua L Payne, Andreas Wagner

**Affiliations:** 1 University of Zurich, Zurich, Switzerland; 2 Swiss Institute of Bioinformatics, Lausanne, Switzerland; 3 The Santa Fe Institute, Santa Fe, USA

**Keywords:** Exaptation, Genotype-phenotype map, Multifunctionality

## Abstract

**Background:**

Latent phenotypes are non-adaptive byproducts of adaptive phenotypes. They exist in biological systems as different as promiscuous enzymes and genome-scale metabolic reaction networks, and can give rise to evolutionary adaptations and innovations. We know little about their prevalence in the gene expression phenotypes of regulatory circuits, important sources of evolutionary innovations.

**Results:**

Here, we study a space of more than sixteen million three-gene model regulatory circuits, where each circuit is represented by a genotype, and has one or more functions embodied in one or more gene expression phenotypes. We find that the majority of circuits with single functions have latent expression phenotypes. Moreover, the set of circuits with a given spectrum of functions has a repertoire of latent phenotypes that is much larger than that of any one circuit. Most of this latent repertoire can be easily accessed through a series of small genetic changes that preserve a circuit’s main functions. Both circuits and gene expression phenotypes that are robust to genetic change are associated with a greater number of latent phenotypes.

**Conclusions:**

Our observations suggest that latent phenotypes are pervasive in regulatory circuits, and may thus be an important source of evolutionary adaptations and innovations involving gene regulation.

## Background

Understanding the origin of novel and beneficial traits — evolutionary adaptations and innovations — is a challenge central to biology. An underappreciated source of such traits are latent phenotypes, by-products of already existing adaptive phenotypes. While themselves neither adaptive nor maintained by natural selection, such latent phenotypes can become sources of novel adaptations in the right environment. In other words, they can become exaptations [1,2]. Prominent examples of latent phenotypes are those of promiscuous enzymes [3], which have one primary, adaptive catalytic activity, and one or more latent activities. For example, in *Escherichia coli* the primary activity of the enzyme aspartate aminotransferase is to transaminate dicarboxylic substrates. However, this enzyme can also transaminate tyrosine and phenylalanine, side-activities that can be increased by over 100-fold via directed evolution [4]. Such promiscuous enzymes are not rare. For example, of 1081 enzymes analyzed in *E. coli*, 37% were found to be promiscuous [5]. Latent phenotypes may also exist in RNA, which can form multiple secondary structures through thermal motion [6,7], as well as in metabolic networks of chemical reactions, which can be viable on carbon sources without prior selection for such viability [8,9].

An important source of evolutionary adaptations are gene regulatory circuits. They are responsible for innovations as diverse as the eyespots of butterflies [10], the vertebrate limb [11], and the body plan of insects [12]. Part of the reason is that these circuits have functions in various processes of embryonic development and physiology, including the interpretation of morphogen gradients during embryogenesis in fruit flies [13], chemotaxis in bacteria [14], and circadian rhythms in organisms as different as fungi and mice [15]. Such functions are embodied in a circuit’s spatiotemporal gene expression phenotype and can often be realized by a large number of different circuits [13,16].

Gene regulatory circuits are often multifunctional. Examples include the segment polarity network in *Drosophila melanogaster*, which guides several distinct developmental processes, including denticle patterning and the specification of neuroblasts [17], the circuit controlling antitoxin production in *E. coli*, which mediates the cellular state between growth and dormancy [18], the lysis-lysogeny switch of bacteriophage lambda, which determines the viral life cycle [19], and the white-opaque switch of *Candida albicans*, which governs numerous cellular phenotypes including metabolic preferences and pathogenicity [20]. Experimental [21] and theoretical [22] studies hint that gene regulatory circuits harbor latent phenotypes because their gene expression patterns often vary when a circuit is exposed to distinct external stimuli. For example, natural [23,24] and synthetic [25,26] regulatory circuits can produce unique gene expression patterns in response to distinct combinations of signals endogenous to a cell. Similarly, the equilibrium expression patterns of model regulatory circuits often differ between initial conditions [22,27].

Experimental work can provide examples of latent phenotypes that exist in any one circuit, but cannot elucidate how frequent latent phenotypes are in gene regulatory circuits in general. This can only be achieved by examining a large number of circuits, a task that is not feasible using existing experimental technologies [28]. Thus, any such work must rely on computational models. Here, we study the incidence of latent gene expression phenotypes in a broad class of model gene regulatory circuits that are highly successful in capturing biological phenomena [29-34]. These circuit models comprise small sets of genes whose ability to turn one another on and off in response to external stimuli is determined by a circuit *genotype*. The regulatory interactions specified by this genotype allow a circuit to form different patterns of gene expression that constitute a circuit’s phenotype(s) and embody its function(s).

Specifically, we here build upon our previous work, which exhaustively characterized the gene expression phenotypes (functions) of all 16,777,216 possible three-gene circuits [35]. This work uncovered several salient features of circuit multifunctionality. First, multiple different circuits — a large *genotype set* of circuits — are capable of performing any given number of *k* functions, and the size of this set decreases exponentially as *k* increases. Second, the genotype set of circuits with any single function always forms a single connected network of genotypes — a *genotype network* — in the space of all circuits, implying that these circuits are reachable from one another via a series of small genotypic changes that preserve circuit function. Third, genotype sets of circuits with more than one function typically fragment into several genotype networks that are disconnected, indicating that such circuits are often mutationally isolated from one another. These observations provide insight into how multifunctionality constrains the size of genotype sets and affects the ability to evolve additional functions, but they do not speak to the existence of latent gene expression phenotypes in these circuits. We here quantify such latent phenotypes exhaustively and answer several questions about them. How many (if any) latent phenotypes do monofunctional circuits harbor? How many latent phenotypes are collectively harbored by a set of circuits with a given number of functions? Does a circuit’s location in a genotype network indicate the number of latent phenotypes it has?

## Results

### The model

**Figure 1:**
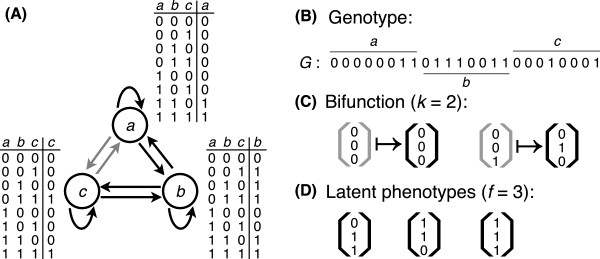
**Schematic illustration of a Boolean circuit.(A)** Each circuit has *N*=3 genes, shown as labeled open circles (*a*,*b*,*c*). Genes can be in one of two states: expressed (1) or not expressed (0). Regulatory interactions are depicted as directed edges *a*→*b*, which indicate that the gene product of *a* regulates the expression of *b*. The signal-integration logic of each gene is captured in a lookup table, where each entry encodes the gene’s regulatory response to one of the 2^*N*^ possible combinations of the states of its regulating gene products. These lookup tables also specify the circuit’s wiring diagram. For example, the expression of gene *a* is independent of gene *c* because its lookup table specifies the Boolean logic function “*a* and *b*.” The regulatory interaction *c*→*a* is thus inactive, as indicated by the gray color of this edge. **(B)** By concatenating the rightmost columns of each lookup table, the signal-integration logic and wiring diagram of a circuit can be represented as a single vector *G* of length *L* = *N* × 2^*N*^. We consider *G* to be the circuit’s genotype. **(C)** The circuit shown in panel (A) is a member of the genotype set of the bifunction *F*^(1)^:〈0,0,0〉↦〈0,0,0〉, *F*^(2)^:〈0,0,1〉↦〈0,1,0〉. **(D)** Each circuit with a given multifunction may map initial states that are not part of the multifunction to new equilibrium states. We consider these to be latent phenotypes. For example, of the five states that are not part of the multifunction shown in (C), three map to the new equilibrium expression states shown in (D), while the other two map to equilibrium expression states that are already part of the multifunction. This circuit therefore has *f* = 3 latent phenotypes, and each is a fixed-point. Other circuits with this bifunction may have more or fewer latent phenotypes.

The model circuits we consider have *N* = 3 genes (Figure 1A) and are encoded by a genotype that specifies both the topology or “wiring” of the circuit and each gene’s *signal-integration logic*, i.e., how the gene’s regulatory region integrates signals from other genes to determine the gene’s expression state. We represent this genotype with a binary genotype vector *G* of length *L* = *N* × 2^*N*^ (Figure 1B). One can think of changes in *G* as mutations in the *cis*-regulatory regions that determine a circuit’s topology and signal-integration logic [23,36]. They include mutations that alter the affinity of a transcription factor binding site, its distance from the transcription start site or from another transcription factor binding site, as well as mutations that result in the gain or loss of a regulatory interaction [37]. Such mutations may lead to changes in a circuit’s interpretation of the regulatory state of the cell, thus altering the circuit’s gene expression pattern [38-43].

Genes in our model circuits can be in one of two states, expressed (1) or not expressed (0). Each circuit is initialized with an expression state *S*_0_, which represents regulatory influences from outside of the circuit, such as signals from the environment or from a higher level in an organism’s regulatory hierarchy [44]. Starting from this initial state, the expression state of each gene can change through the influence of the expression states of its regulating gene products and its signal-integration logic. The circuit’s gene expression state eventually converges on an equilibrium gene expression pattern *S*_*∞*_ with period *p*, which can be a fixed-point (*p* = 1) or periodic (*p* > 1).

Regulatory circuits that control developmental and physiological processes often do so via fixed-point gene expression patterns [17]. Examples include the circuits controlling the interpretation of morphogen gradients in the early *Drosophila* embryo [45] and floral organ specification in *Arabidopsis thaliana*[30]. We thus consider the phenotype or *function* of a circuit to be an initial state paired with the fixed-point equilibrium state that a circuit attains through its gene expression dynamics when starting from this initial state, i.e., *F* = (*S*_0_,*S*_*∞*_) (Figure 1C). Since there are 2^*N*^ distinct initial states, a circuit can have up to 2^*N*^ such functions *F*^(1)^…*F*^(*k*)^, as long as each initial state maps uniquely to a fixed-point equilibrium expression state ($S_{\infty }^{\left(i\right)}\neq S_{\infty }^{\left(j\right)}$ for all *i*,*j*). In the parlance of dynamical systems theory, such circuits can be said to display multistability on a subset of the state space.

Despite its many abstractions, this modeling framework has provided important insight into both general and specific properties of gene regulatory circuits. For example, it has been used to understand the influence of various facets of circuit topology on the robustness of gene expression patterns to genetic perturbations [27,46,47] and on the ability of circuits to adapt to novel environmental conditions [48,49]. Moreover, variants of the model have been used to predict the expression dynamics of the genes involved in (i) specifying the endomesoderm and skeletogenic spatial domains in the embryo of the sea urchin *Strongylocentrotus purpuratus*[32], (ii) modulating the expression of the tumor suppressing protein p53 in human breast cancer cells [33], and (iii) controlling circadian oscillations in the fungus *Neurospora crassa* and the plant *A. thaliana*[34]. We choose Boolean logic circuits as our modeling framework because of their ability to capture both general and specific properties of gene regulatory circuits and because they allow us to study and alter both a circuit’s wiring diagram and its signal-integration logic.

Circuits with multiple phenotypes or functions are not unusual. For example, the circuit controlling the lysis-lysogeny decision in bacteriophage lambda is bifunctional, while the circuit controlling the patterning of the neural tube in vertebrates is trifunctional, because it produces three spatially distinct ventral progenitor domains [50]. It is important to note that our knowledge of multifunctionality in biological circuits is limited to those conditions that have been tested experimentally, which most likely represent only a subset of the environmental and regulatory cues experienced by a circuit. The preceding estimates of multifunctionality in biological circuits should therefore be considered as a lower bound. In contrast to biological circuits, it is possible to comprehensively probe the functionality of synthetic circuits. Examples where this has been accomplished include the bifunctional circuit that selectively induces apoptosis in cancer cells [51] and the quadrifunctional circuit that serves as a combinatorial “decoder” in human embryonic kidney cells [52]. We here use the terms *monofunction*, *bifunction*, *trifunction*, etc. to indicate the number of functions in a circuit. More generally, we call any set of more than one function a *multifunction* and a specific set of *k* functions a *k-function*.

Multiple circuit genotypes may have a given *k*-function, and we refer to the collection of such circuits as a *genotype set*, which may comprise one or more *genotype networks*[35]. In addition to its *k*-function, each circuit in a genotype set may also have one or more *latent phenotypes*, potential exaptations which we define as an equilibrium expression pattern *S*_*∞*_ that is not part of the circuit’s *k*-function (Figure 1D). We do not require the latent phenotype to be a fixed-point expression pattern (*p* = 1) nor do we require it to be realized under a specific initial condition *S*_0_, because it is not possible to determine *a priori* which form of expression pattern (fixed-point or periodic) might become beneficial for the survival of the organism, nor which initial condition might exist to facilitate its formation (in the supporting online material, we consider the case where latent phenotypes are required to be fixed-point expression states). Biologically important non-fixed-point equilibrium expression patterns include the genetic oscillators that control circadian rhythms [15], segmentation clocks [53], and the cell cycle [54].

In a previous contribution, we exhaustively enumerated the genotype-phenotype (function) map of all 2^*L*^ = 2^24^ = 16,777,216 possible circuit genotypes for all 2^*N*^ = 8 initial states of circuits with *N* = 3 genes, revealing a total of 32,399 unique *k*-functions [35]. Here, we study the circuits with these *k*-functions in more detail, focusing on their latent phenotypes.

### Monofunctional circuits typically have latent phenotypes

**Figure 2:**
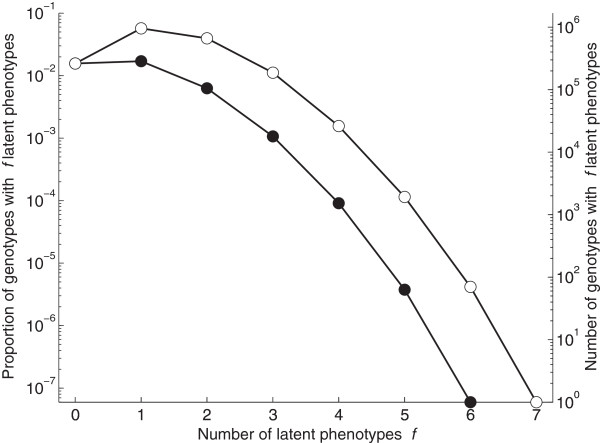
**Monofunctional circuits typically have latent phenotypes.** Each data point depicts the proportion and number of monofunctional circuits with *f* latent phenotypes. The white and black circles correspond to an identity (e.g., 〈0,0,0〉↦〈0,0,0〉) and transition (e.g., 〈0,0,0〉↦〈0,0,1〉) mapping, respectively. The genotype set of each identity mapping comprises 2,097,152 genotypes, while that of each transition mapping comprises 672,592 genotypes [35]. All 8 identity and 56 transition monofunctions exhibit a pattern exactly identical to that of the white and black circles, respectively, shown in the figure. The lines are provided as visual guides. Note the logarithmic scale of the y-axis.

There are 64 possible monofunctions, and we previously found that these monofunctions exhibit two prominent features [35]: Their genotype sets comprise many (> 10^5^) genotypes that are part of a single genotype network and the number of genotypes in this network is independent of the specific monofunction — it depends only on whether the initial and equilibrium states are the same (i.e., an *identity mapping*, *S*_0_ = *S*_*∞*_) or different (i.e., a *transition mapping*, *S*_0_ ≠ *S*_*∞*_). Building on this work, we analyzed each of the 64 possible monofunctions systematically, and first asked whether their constituent genotypes had latent phenotypes and if so, how many. To answer these questions, we calculated the number of latent phenotypes *f* per genotype, which we computed as the number of new equilibrium expression patterns *S*_*∞*_ the genotype can realize under initial conditions that are not part of its function (Figure 1D; Methods). Figure 2 shows the proportion and number of genotypes with *f* latent phenotypes among circuits with the two types of monofunctions. Remarkably, for both types of monofunctions, there are more genotypes with one latent phenotype than with no latent phenotypes. Overall, 88% percent of circuits with an identity mapping and 61% percent of circuits with a transition mapping have at least one latent phenotype. In other words, latent phenotypes are the rule rather than the exception among monofunctional circuits. While the number of genotypes with *f* latent phenotypes decreases exponentially as *f* increases (Figure 2), there are hundreds of thousands of circuits with more than one latent phenotype. Additional file 1: Figure S1 shows a nearly identical trend for latent fixed-point phenotypes. To illustrate the types of latent phenotypes that are observed in these circuits, Additional file 1: Figure S2 shows the proportion of latent phenotypes that are fixed-point and periodic for identity and transition monofunctions.

Next we determined how many latent phenotypes were harbored in the genotype sets of the 64 possible monofunctions. Specifically, we considered *all* of the circuits with a given monofunction and calculated the size of their *latent repertoire*, i.e., the number of unique latent phenotypes that these circuits collectively harbor. We found that the size of a monofunction’s latent repertoire depends only on the type of monofunction (i.e., identity vs. transition mapping). Of the 16,072 possible equilibrium expression patterns of three-gene circuits (Methods), circuits with any one identity mapping had a latent repertoire of 2372 (14.8%) phenotypes, whereas circuits with a transition mapping had a latent repertoire of 415 (2.6%) phenotypes. In the supporting online material, we provide a combinatorial explanation for the observation that latent repertoire size depends only on the type of monofunction. We emphasize that all circuits with a given monofunction are accessible from one another through a series of small genotypic changes that do not affect the monofunction, because the genotype set of each monofunction consists of a single connected genotype network [35]. This means that all latent phenotypes accessible from some circuit with a given monofunction are reachable through gradual evolutionary change.

Finally, we determined the extent to which latent repertoires varied among monofunctions. To do this, we considered each possible pairing of monofunctions and calculated the number of latent phenotypes that were present in the intersection and union of their latent repertoires. We then divided the former number by the latter to provide a fractional measure of similarity between latent repertoires. We found that this fraction is typically very small (< 0.2), meaning that monofunctions share few latent phenotypes (Additional file 1: Figure S3). This indicates that a circuit’s primary function severely constrains the spectrum of accessible latent phenotypes.

### Latent repertoire size increases with genotype set size

**Figure 3:**
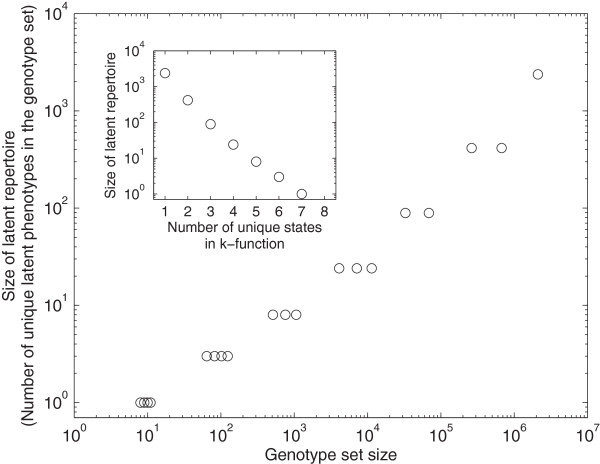
**Latent repertoire size increases with genotype set size and depends solely upon the number of unique states in a*k*-function.** The size of each *k*-function’s latent repertoire is shown in relation to the size of its genotype set. The inset shows the relationship between a *k*-function’s latent repertoire size and the number of unique states in the *k*-function. Note the logarithmic scale of both axes in the main panel and the y-axis of the inset; these conceal the data for the sole 8-function, which has no latent phenotypes and a genotype set size of one.

We have found that the latent repertoire size increases with genotype set size for monofunctions. We next asked whether this is also the case for multifunctional circuits, i.e., we extended our analysis to include all 32,399 *k*-functions. Figure 3 shows that *k*-functions with larger genotype sets also have larger latent repertoires (Spearman’s *r* = 0.98, *p*<1 × 10^-50^). For example, the smallest genotype sets contain only a single genotype and these have no latent phenotypes, while the largest genotype set has over two million genotypes, and these circuits collectively have thousands of latent phenotypes. Notably, every *k*-function with more than one circuit in its genotype set has at least one latent phenotype. Thus, while it is possible that an individual circuit does not have a latent capacity for exaptation (Figure 2), there always exists another circuit with the same *k*-function that does have such a capacity, so long as the *k*-function’s genotype set comprises more than one circuit. Similar results are observed for latent fixed-point phenotypes, although the latent repertoire sizes are necessarily reduced (Additional file 1: Figure S4). To further illustrate the properties of latent phenotypes, Additional file 1: Figure S5 shows the number of transient states encountered in the trajectory from initial to equilibrium state.

The step-like shape of the trend in Figure 3 hints that the latent repertoire size may depend on qualitative features of a *k*-function. Indeed, the inset of Figure 3 shows that the number of unique states in the *k*-function uniquely determines latent repertoire size. For example, the latent repertoire sizes of the monofunction *F*^(1)^:〈0,0,0〉↦〈0,0,1〉 and the bifunction *F*^(1)^:〈0,0,0〉↦〈0,0,0〉, *F*^(2)^:〈0,0,1〉↦〈0,0,1〉 are identical, as these two *k*-functions comprise the same expression states. Moreover, these repertoires contain the same latent phenotypes, despite the two corresponding *k*-functions comprising distinct genotype sets. This pattern is apparent in any pair of *k*-functions that comprise the same set of expression states, as shown analytically in the supporting online material.

### Latent phenotypes vary within and among the genotype networks of a *k*-function

**Figure 4:**
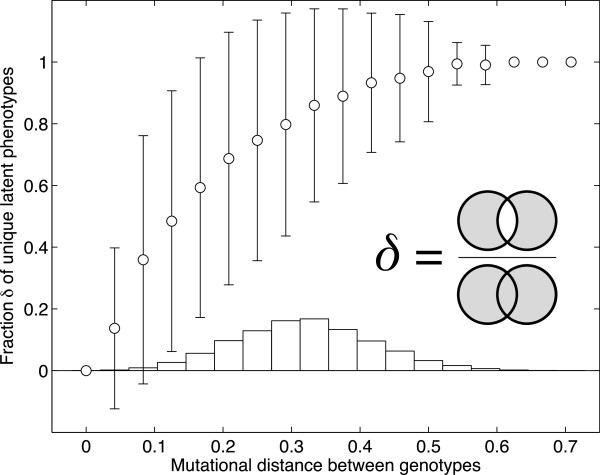
**Latent phenotypes vary within the dominant genotype network of a*k*-function.** The data shown is based on 100,000 sampled pairs of genotypes from the dominant genotype network of the bifunction *F*^(1)^:〈0,0,0〉↦〈0,0,1〉, *F*^(2)^:〈0,1,0〉↦〈0,1,1〉. Open circles depict the mean fraction *δ* of latent phenotypes that are unique to one genotype or the other in each pair (see inset and Methods, Eq. 3), shown in relation to the mutational distance between these genotypes. Error bars correspond to one standard deviation. The histogram shows the distribution of sampled mutational distances between circuits in a pair.

We have shown that the size of a *k*-function’s latent repertoire depends only on the number of unique expression states that occur in the *k*-function. However, we have not addressed how these latent phenotypes vary amongst the individual genotypes that have the same *k*-function. We therefore next asked whether genotypes that are separated by a small number of mutations are likely to have more similar latent phenotypes than those separated by many mutations. To answer this question, we sampled 100,000 pairs of genotypes from the largest connected genotype network (i.e., the dominant genotype network) of every *k*-function. For each pair of genotypes, we determined (i) their mutational distance from one another, and (ii) the fraction *δ* of latent phenotypes that is unique to one genotype or the other (Figure 4, inset; Methods). If *δ* increases with the mutational distance between genotypes, then a circuit’s location on a genotype network determines its latent capacity for exaptation.

Figure 4a shows *δ* in relation to the mutational distance between genotypes on the dominant genotype network of a representative bifunction. The fraction of unique phenotypes increases with mutational distance (Spearman’s *r* = 0.35, *p* < 1 × 10^-50^), indicating that two genotypes separated by many mutations are more likely to have latent phenotypes that differ from one another than two genotypes separated by few mutations. Such a positive association also exists for the other *k*-functions, as long as the size of their latent repertoire is greater than one (Additional file 1: Figure S6). These results show that a circuit’s location on a genotype network influences which latent phenotypes are available to it, an observation that also applies to latent fixed-point phenotypes (Additional file 1: Figure S7).

Many multifunctions have fragmented genotype sets, i.e., they consist of multiple disconnected genotype networks [35]. To understand how genotype set fragmentation may constrain the number of available latent phenotypes, we next asked how latent phenotypes vary between the genotype networks of fragmented genotype sets. First, for each *k*-function with a fragmented genotype set, we calculated the number of unique latent phenotypes found in the dominant genotype network, but not in the *peripheral* genotype networks (i.e., those different from the dominant genotype network). We found that for most *k*-functions the majority of latent phenotypes were only realized by circuits in the dominant genotype network (Additional file 1: Figure S8). For example, the median fraction (among all *k*-functions with a fragmented genotype set) of the latent repertoire that is accessible only from the dominant genotype network is 0.71. Second, we calculated the number of unique latent phenotypes that were present in the peripheral genotype networks, but not in the dominant genotype network. Of all the *k*-functions with fragmented genotype sets, there was not a single such latent phenotype. Since only a minority of circuits belong to the peripheral genotype networks of any *k*-function with a fragmented genotype set (the median fraction is 18%; Additional file 1: Figure S9), these results suggest that genotype set fragmentation does not impose severe constraints upon the accessibility of latent phenotypes. Finally, we found that the number of unique latent phenotypes per genotype network increased with the size of the genotype network (Additional file 1: Figure S10), similar to the association observed between latent repertoire size and genotype set size (Figure 3).

### Robust circuits have many latent phenotypes

Studies of systems as diverse as RNA [55] and programmable hardware [56] have shown that a genotype’s robustness to genetic change is inversely correlated with its potential to bring forth novel phenotypes via mutation. In previous work, we also observed this inverse correlation in the model regulatory circuits considered here [57]. We therefore next asked whether a circuit’s mutational robustness — measured as its number of neighbors with the same *k*-function (i.e., number of connections in a genotype network) — is inversely correlated with its number of latent phenotypes. To answer this question, we focused solely on multifunctions composed of at least one transition mapping, because only circuits with such multifunctions exhibit variability in their mutational robustness [35].

**Figure 5:**
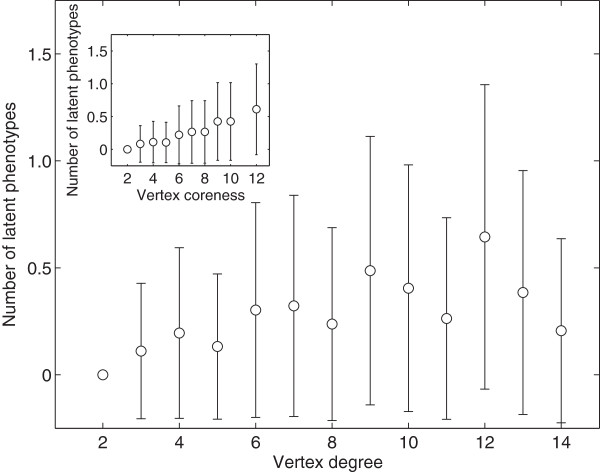
**Robust genotypes have many latent phenotypes.** Data are based on the dominant genotype network of the same bifunction as in Figure 4. Open circles depict the mean number of latent phenotypes among all genotypes with a given vertex degree (main panel) or coreness (inset). Error bars correspond to a single standard deviation.

Figure 5 shows that a genotype’s vertex degree and its number of latent phenotypes are weakly, but significantly positively correlated (Spearman’s *r* = 0.13, *p*<1.2 × 10^-41^), indicating that mutationally robust circuits have an increased capacity for exaptation. Intriguingly, an additional measure of mutational robustness — vertex coreness (Methods) — shows a stronger and more consistent association with a circuit’s number of latent phenotypes (Spearman’s *r* = 0.25, *p*<1 × 10^-50^; Figure 5, inset; Additional file 1: Figure S11). Nearly identical trends are observed for latent fixed-point phenotypes, although the strength of the correlation is reduced (Additional file 1: Figure S12). These positive correlations between a genotype’s number of latent phenotypes and the degree and coreness of its corresponding vertex in a genotype network also hold for other multifunctions, so long as the genotype set size is sufficiently large (Additional file 1: Figure S11). In sum, mutational robustness generally does not hinder, but rather facilitates, latent phenotypes in gene regulatory circuits.

### Latent repertoire size increases with the number of genes in a circuit

Our focus on small, three-gene circuits facilitated an exhaustive analysis of genotype space. However, regulatory circuits often comprise more than three genes (e.g., [58-60]) and are typically part of larger regulatory networks [44]. As such, it is important to understand how our observations might translate to larger circuits. To do so, we randomly sampled 10,000 genotypes of monofunctional circuits with 3 ≤ *N* ≤ 10 genes and determined the average number of latent phenotypes per circuit. (We note that the analogous sampling procedure for multifunctional circuits is precluded by the drastically reduced number of circuits with *k* > 1 functions [35].) We found that the average number of latent functions per monofunctional circuit increases linearly in *N* and only very gradually, such that a circuit with *N* = 10 genes has roughly twice as many latent phenotypes as a circuit with *N* = 3 genes (Additional file 1: Figure S13A). This indicates that the proportion of possible latent phenotypes that an individual circuit can realize decreases exponentially in *N* (Additional file 1: Figure S13B), since the maximum number of latent phenotypes in a circuit with *k* functions is 2^*N*^-*k*. Nevertheless, we expect that the size of a multifunction’s latent repertoire will increase with *N*, for the following reasons. First, as *N* increases, the number of possible latent phenotypes increases exponentially (see Eq. 2 in Methods) and genotype set size increases hyperexponentially [35]. Second, these genotype sets comprise large dominant genotype networks of dissimilar circuits [57], which will typically have different latent phenotypes (Figure 4). Taken together, these observations indicate that the size of a multifunction’s latent repertoire will increase with the number of genes *N* in the circuit despite the decrease in the proportion of latent phenotypes realized by individual circuits.

## Discussion

We have exhaustively characterized the latent phenotypes of model three-gene regulatory circuits. While individual circuits typically have few latent phenotypes, the entire set of circuits (genotypes) with a given set of functions (a multifunction) collectively has many latent phenotypes, which constitute the multifunction’s latent repertoire. This latent repertoire is a source of potential novel adaptations in gene expression patterns, because most circuits in a genotype set are part of a single genotype network, and each latent phenotype in the repertoire can be realized by one of these circuits. Thus, a circuit with any phenotype in the latent repertoire can evolve via a series of mutations that preserve the circuit’s functions.

Previous work on systems ranging from RNA [61] to metabolic networks [62] shows that genotype networks (also called neutral networks [63]) can facilitate the origin of novel and beneficial phenotypes. By permeating the space of possible genotypes, they allow for the accumulation of genetic diversity in evolving populations while permitting the preservation of an existing phenotype [64]. In doing so, they provide mutational access to genotypes with novel phenotypes [55,65]. Our work suggests two additional reasons why genotype networks facilitate the origin of novel phenotypes. First, the latent repertoire of an entire genotype network is usually greater than that of a single circuit genotype. This means that the very existence of genotype networks enables the origin of novel circuit functions from latent gene expression phenotypes. Second, the size of a multifunction’s latent repertoire increases with the size of its genotype set. This means that regulatory functions (gene expression patterns) that can be realized by greater numbers of circuits — which usually also form large genotype networks — harbor a greater potential for evolutionary innovation originating from latent phenotypes. Since the size of the genotype set associated with a given phenotype can be used as a proxy for the phenotype’s robustness to genetic change [55,57], those circuits with highly robust gene expression phenotypes can explore a greater variety of latent phenotypes.

We also investigated the relationship between the mutational robustness of individual circuit genotypes and their number of latent phenotypes, uncovering a positive correlation between these two properties. Robust genotypes therefore have an increased capacity for exaptation. This is an intriguing observation because theoretical results suggest that such genotypes are likely to appear in neutrally evolving populations [66]. Thus, in contrast with adaptations that arise via mutation — where genotypic robustness hinders, but phenotypic robustness facilitates, the origin of novel phenotypes [67] — adaptations that arise via latent phenotypes are facilitated by both genotypic and phenotypic robustness.

The genotype sets of multifunctions are often fragmented into several isolated genotype networks [35]. Such fragmentation is not unique to regulatory circuits. It has been observed in models of RNA secondary structure [68] and protein quaternary structure [69]. Here, we found that larger genotype networks harbored more latent phenotypes than smaller genotype networks, indicating that a circuit’s latent capacity for exaptation is dependent upon which genotype network the circuit belongs to. Because we know very little about the genotype networks of biological circuits, we cannot say whether the enhanced capacity for exaptation that is conferred by a large genotype network could be realized by extant biological circuits. We anticipate that the genotype network membership of biological circuits will be affected by a variety of factors, including historical contingency [70], designability [71], and robustness to environmental and genetic perturbations [72]. As screening and selection strategies for gene circuits continue to advance [28], it may become possible to experimentally tease apart the relative influences of these various factors.

One caveat of using a Boolean model of gene regulatory circuits is that the number of mutations required to transition from one logical function to another may not directly correspond to the number of mutations required for the same transition in a biological context. For example, in our model, mutating a gene’s signal-integration logic from OR to AND requires twice as many mutations as the transition from OR to XOR. While theoretical work suggests that the former transition would indeed require several mutations [73], both theoretical [73] and experimental work [74-76] indicate that XOR logic is exceedingly difficult to implement, suggesting that the latter transition would require a greater number of mutations. Moreover, such mutational transitions are often accompanied by the addition and subsequent deletion of edges that are not involved in the logical function that is being mutated. Some of these issues may be overcome using biophysical models of regulatory circuits, which employ experimentally derived TF binding preferences [77] or TF-DNA crystal structures [78] to describe a TF’s binding affinity to short DNA sequences embedded within longer promoter sequences. However, the length of the promoter sequences considered in such models (e.g., between 50 and 300 base pair per gene in [78]) preclude the exhaustive enumeration of the space of regulatory circuits, and would therefore render the analyses considered here infeasible.

Our results suggest that latent phenotypes pervade gene regulatory circuits and may therefore contribute to the origin of adaptations and evolutionary innovations. Anecdotal evidence supporting this view comes from comparative genomics studies of the redeployment of ancient transcriptional regulatory circuits in novel spatial domains. For example, vertebrate dentitions first appeared in the pharynx of jawless fishes. The regulatory circuit that controlled the development of pharyngeal teeth was subsequently redeployed to control the development of oral dentitions [79]. Similarly, the regulatory circuit that controls the patterning of the insect wing blade was redeployed in butterflies for the function of generating eyespots, a predator avoidance strategy that evolved much later than the insect body plan [10]. In both cases, the alteration of the spatial domain of an existing gene expression pattern formed the basis of an evolutionary innovation. While it is not known whether the ancestral circuits exhibited latent phenotypes in their novel spatial domains or whether the circuit redeployment evolved *de novo*, recent evidence from enhancer evolution in fruit flies suggests that the latent expression phenotypes of gene regulatory circuits can become exaptations [80]. Specifically, expression of the gene *Neprilysin-1* in the developing visual system of *Drospophila santomea* derives from the co-option of enhancer sequences active in other tissues that exhibited latent activity in the optic lobes of the last common ancestor of *D. santomea* and *D. yakuba*. Thus, the latent phenotypes of regulatory circuits can provide a foundation for evolutionary adaptations and innovations.

Classical exaptations — adaptive traits with non-adaptive origins — include the feathers of birds [81] and the Panda’s thumb [1], which originated as a wrist-bone. Such traits usually need to be modified to assume a new function. In contrast, a latent phenotype already embodies this new role as a by-product of a system’s existing function. Once this phenotype becomes favored by natural selection, the system is already poised for exaptation. An intriguing question for future work is whether adaptations that originated from latent phenotypes are prevalent or rare among exaptations.

## Conclusions

Together with previous work on systems as different as metabolism [9], enzymes [5], and RNA [7], our observations suggest that latent phenotypes are ubiquitous in biological systems and could thus be a common source of new adaptations.

## Methods

### Model details

The Boolean circuits we consider have *N* = 3 genes. The expression state of each gene can in principle be influenced by any other gene. Each gene *i* is associated with a deterministic and synchronous update function *ϕ*_*i*_ that dictates how its binary expression state *σ*_*i*_(*t*) at time *t* will respond to the expression states of the other genes in the circuit at time *t*-1:

(1) $$\sigma_{i}(t)=\phi_{i}(\sigma_{i}(t-1),\sigma_{j}(t-1),\sigma_{k}(t-1)).$$

The update function *ϕ*_*i*_ constitutes the gene’s signal-integration logic and can be represented as a look-up table that maps all of the 2^*N*^ possible combinations of input expression states to an output expression state (Figure 1A). The signal-integration logic of the entire circuit can be represented compactly as a vector *G* of length *L* = *N* × 2^*N*^, constructed by concatenating the output columns of all *N* look-up tables (Figure 1B). It is easy to see that a circuit’s signal-integration logic also encodes its “wiring” diagram, because it specifies whether one gene’s expression state is influenced by another gene or not (e.g., gene *c* is not influenced by gene *a* in Figure 1A). The vector *G* thus provides a complete description of the circuit. Because this vector is ultimately encoded in a genotype, we also refer to it as such.

The dynamics of the circuit begin with an initial expression state *S*_0_. At each time step *t*, the circuit’s gene expression states are updated synchronously according to *G*, forming a “trajectory” through state space (e.g., *S*_0_ → *S*_*t*_ → *S*_*t*+1_ → …). Since the updates are deterministic and the number of genes is finite, this trajectory will eventually converge on an equilibrium expression pattern *S*_*∞*_, which may comprise one or more states, i.e., it could be a cycle with some period *p* > 1 or a fixed-point (*p* = 1).

### Constructing the genotype networks of a *k*-function

We refer to the set of circuits with a given *k*-function as a genotype set. For Boolean circuits with *N* = 3 genes, there are a total of 32,399 unique *k*-functions, each with its own genotype set [35]. We emphasize that a genotype *G* may be a member of more than one genotype set. For each *k*-function, we determined the number of “mutations” that separated each possible pair of circuits in a genotype set by calculating the Hamming distance of their genotype vectors. We created genotype networks by representing each of these genotypes as a vertex and connecting pairs of vertices with an edge if their corresponding genotypes were separated by a single mutation (i.e., a Hamming distance of 1).

### Number of equilibrium expression patterns

There are 16,072 possible equilibrium expression patterns *S*_*∞*_ for a circuit with *N* = 3 genes. This number is easily calculated by summing over the number of possible equilibrium expression patterns of each period 1 ≤ *p* ≤ 2^*N*^, as follows. When *p* = 1, there are 2^*N*^ choices for *S*_*∞*_. When *p* = 2, there are 2^*N*^ × (2^*N*^-1)/2 choices for *S*_*∞*_, where the numerator counts the number of possible permutations of 2 states chosen from 8 and the denominator corrects for the number of these permutations that are cyclically equivalent (i.e., *S*_*∞*_ : *S*_*a*_ → *S*_*b*_ is the same as *S*_*∞*_ : *S*_*b*_ → *S*_*a*_). This reasoning can be extended to any *p* with 1 ≤ *p* ≤ 2^*N*^, and by summing up over the number of possible equilibrium expression for each *p* one obtains:

(2) $$|\{S_{\infty }\}|=\overset{2^{N}}{\underset{p=1}{\sum }}\frac{1}{p}\left(\frac{2^{N}!}{(2^{N}-p)!}\right).$$

### Number of latent phenotypes

For each circuit in the genotype set of each *k*-function, we determined the circuit’s number of latent phenotypes *f* as follows. For each initial state *S*_0_ that was not already part of the *k*-function, we determined the circuit’s equilibrium expression pattern *S*_*∞*_. If this equilibrium expression pattern was distinct from any of those in the *k*-function, then it was considered a latent phenotype. The total number of unique equilibrium expression patterns arrived at in this manner was then taken as the circuit’s number of latent phenotypes *f*. For example, the bifunction shown in Figure 1C includes a total of three states (〈0,0,0〉, 〈0,0,1〉, 〈0,1,0〉). We therefore initialized the circuit shown in Figure 1A with each of the five remaining states to determine *f*. Of these five initial states, three led to the new equilibrium expression patterns shown in Figure 1D. The number of latent phenotypes for this circuit is therefore three and each is a fixed-point (*p* = 1) phenotype.

### Coreness calculation

We determined the coreness *c*[82] of a vertex using the following iterative pruning procedure. First, we identified all vertices with degree *d* = 1 and removed them from the genotype network, along with the edges incident to them. If, as a result of this pruning, any remaining vertices had degree *d* = 1, then we also removed them and their edges. We repeated this step until there were no vertices with degree *d* = 1. We then assigned all of the vertices removed in this procedure coreness *c* = 1. We then repeated the entire process for vertices with degree *d* = 2, and so on, until no vertices remained in the genotype network, resulting in the assignment of a coreness value to each vertex. Note that we use the term “coreness” rather than the more conventional term “*k*-core” to avoid confusion with our use of the variable *k* in a *k*-function.

### Calculating the fraction of unique latent phenotypes

We calculated the fraction *δ* of unique latent phenotypes between two circuits as

(3) $$\delta =1-\frac{\left|e_{1}\cap e_{2}\right|}{\left|e_{1}\cup e_{2}\right|},$$

where *e*_1_ and *e*_2_ are the sets of latent phenotypes of the two circuits in the pair [83]. When calculating *δ*, we restricted our attention to genotypes with at least one latent phenotype. If *δ* is small for genotypes separated by only a few mutations, but large for genotypes separated by many mutations, this indicates that a genotype’s location in a genotype network influences which latent phenotypes are available to it.

## Competing interests

The authors declare that they have no competing interests.

## Authors’ contributions

JLP and AW conceived and designed the analyses. JLP performed the analyses and analyzed the data. JLP and AW wrote the paper. Both authors read and approved the final manuscript.

## Supplementary Material

Additional file 1 **Supporting online material.** This pdf file contains supplementary results and figures S1–S13. Click here for file
