# Supplementary material for: Latent phenotypes pervade gene regulatory circuits
Source: BMC Syst Biol. 2014 May 30;8:64. doi: 10.1186/1752-0509-8-64 (PMC4061115; doi:10.1186/1752-0509-8-64)
Supplement: Additional file 1 — Supporting online material. This pdf file contains supplementary results and figures S1–S13. [file 1752-0509-8-64-S1.pdf]

SUPPORTING ONLINE MATERIAL FOR

Latent phenotypes pervade gene regulatory circuits

Joshua L. Payne and Andreas Wagner

Full list of author information is available in the main text

Supplementary results

Analytical determination of the latent repertoire size per k-function

Here we analytically determine the number of latent phenotypes harbored by the set of circuits with a given k-function. This number, which we refer to as *latent repertoire size*, depends only upon the number of unique states encountered by each circuit in mapping its initial states to its equilibrium states. As in the main text, we consider circuit functions with fixed-point equilibrium expression states S_∞, but we allow latent phenotypes to be either fixed-point or periodic. We refer to functions with $S_0 = S_\infty$ as *identity functions* and to functions with $S_0 \neq S_\infty$ as *transition functions*. We use the notation $\langle I, T \rangle$ to denote the *composition* of a k-function, where I is the number of identity functions and T is the number of transition functions. We use the variable Z to denote the total number of states in a k-function, $Z = I + 2T$.

We now show that the number of states Z in a k-function uniquely determines latent repertoire size, F (cf. Fig. 3). Since a latent phenotype cannot comprise any of the Z states that are in the k-function and every conceivable combination of functions is realized by at least one circuit in the genotype space considered here [35], the problem of determining a k-function's latent repertoire size is reduced to determining the number of phenotypes that do not contain any of the k-function's Z states, necessitating only a slight modification of Eq. 2 from the main text:

$$F = \sum_{p=1}^{2^N - Z} \frac{1}{p} \frac{(2^N - Z)!}{(2^N - p - Z)!}. \tag{S1.1}$$

This equation explains why, for example, the monofunction $F^{(1)} : \langle 0,0,0 \rangle \mapsto \langle 0,0,1 \rangle$ has the same latent repertoire size as the bifunction $F^{(1)} : \langle 0,0,0 \rangle \mapsto \langle 0,0,0 \rangle$, $F^{(2)} : \langle 0,0,1 \rangle \mapsto \langle 0,0,1 \rangle$: While these functions have different compositions ($\langle 0,1 \rangle$ and $\langle 2,0 \rangle$, respectively), they comprise the same number of total states ($Z = 2$) and therefore have the same latent repertoire size. Moreover, these latent repertoires comprise the same latent phenotypes: all possible equilibrium expression states that do not contain $\langle 0,0,0 \rangle$ or $\langle 0,0,1 \rangle$.

The number of monofunctional circuits with f latent phenotypes depends only upon the composition of the k-function

In Figs. 2 and S1, we showed that the number of monofunctional circuits with f latent phenotypes depends only upon the composition of the monofunction, specifically whether it is an identity or a transition function. Here we explain this finding

via combinatorial enumeration. We show that determining the number of monofunctional circuits with f latent phenotypes is equivalent to determining the number of attractor landscapes with $f+1$ attractors (the $+1$ accounts for the monofunction itself), where *attractor* is defined as an equilibrium expression state (fixed-point or periodic) and *attractor landscape* is defined as the set of all attractors and the sequence of states that lead into those attractors [22], as realized by a single circuit. This equivalence holds because each unique circuit genotype G yields a distinct attractor landscape [35].

To elucidate this equivalence, we consider the simplest possible case of enumerating the number of attractor landscapes with $f+1$ fixed-point attractors and show that this is the same as the number of circuits with an identity monofunction ($k=1$) and f latent fixed-point phenotypes (Fig. S1). Similar enumerations can be made for transition monofunctions, k-functions of arbitrary composition, and latent phenotypes of arbitrary periodicity, though the calculations are more cumbersome.

We use graphical illustrations of attractor landscapes to complement each enumeration. In these illustrations, attractor landscapes are depicted as directed graphs, in which vertices represent circuit states and directed edges denote transitions from one state to another. Self-loops designate states that are fixed-point attractors and a double-lined vertex represents an identity monofunction (e.g., $\langle 0,0,0\rangle \mapsto \langle 0,0,0\rangle$). In characterizing the graphs that represent each attractor landscape, we differentiate between a graph's *isomorphism* and its *labeling*. The former corresponds only to the attractor landscape's topology (i.e., its vertices and edges), while the latter corresponds to the states represented by the vertices.

The number of circuits with 7 latent fixed-point phenotypes

We begin by counting the number of attractor landscapes with 8 fixed-point attractors. Since 7 of these attractors need to be chosen from the 7 states that are not part of the monofunction, there is only one possible isomorphism:

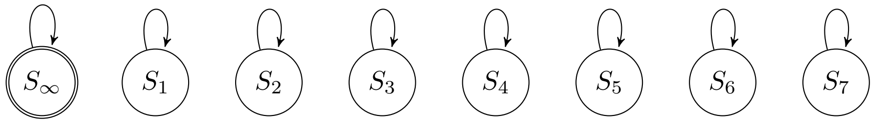

Moreover, there is only one possible labeling of this isomorphism, meaning that there are only

$$\binom{7}{7} \times 1 = 1 \tag{S2.1}$$

attractor landscapes with 7 fixed-point attractors. This number is equivalent to the number of monofunctional circuits with 7 latent fixed-point phenotypes (Fig. S1, open circles).

The number of circuits with 6 latent fixed-point phenotypes

Next we enumerate the number of attractor landscapes with 7 fixed-point attractors. Six of these attractors must be chosen from the seven states that are not part of the monofunction, while the remaining state must transition to one of the 7 fixed-point attractors; otherwise, the remaining state would be an attractor itself. There is therefore only one possible isomorphism:

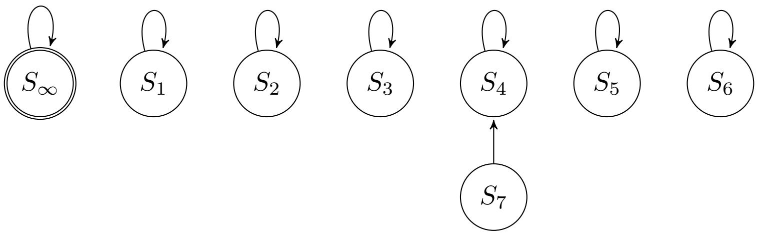

This isomorphism has seven possible labelings, since the state that is not an attractor can transition to any one of the 7 attractors. There are therefore

$$\binom{7}{6} \times 7 = 49 \tag{S3.1}$$

attractor landscapes with 7 fixed-point attractors. This number is equivalent to the number of monofunctional circuits with 6 latent fixed-point phenotypes (Fig. S1, open circles).

The number of circuits with 5 latent fixed-point phenotypes

There are 3 isomorphisms of attractor landscapes with 6 fixed-point attractors, and each has multiple labelings. In each isomorphism, 5 of these attractors must be chosen from the 7 states that are not part of the monofunction. What is done with the remaining 2 states defines the 3 distinct isomorphisms.

In the first isomorphism, the 2 remaining states transition directly to an attractor.

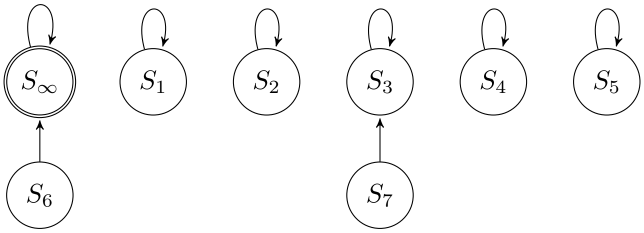

Since both of these states can transition to any one of the 6 attractors, there are

$$\binom{7}{5} \times 6 \times 6 = 756 \tag{S4.1}$$

possible labelings of this isomorphism.

In the second isomorphism, the two remaining states form a linear chain that attaches to one of the 6 attractors.

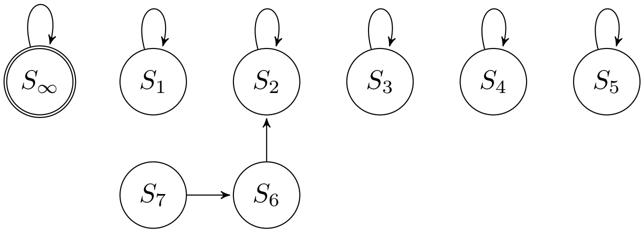

Since this chain can transition to any one of the 6 attractors and can be ordered in 2 distinct ways, there are

$$\binom{7}{5} \times 6 \times 2 = 252 \tag{S4.2}$$

possible labelings of this isomorphism.

In the third isomorphism, the two remaining states form an attractor of period 2.

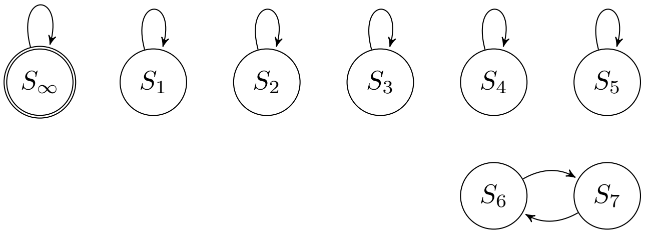

Once the 5 attractors are chosen from the 7 states, there is only one possible way to arrange the periodic attractor. There are therefore

$$\binom{7}{5} \times 1 = 21 \tag{S4.3}$$

possible labelings of this isomorphism. Summing these three numbers gives 1029, which is equivalent to the number of monofunctional circuits with 5 latent fixed-point phenotypes (Fig. S1, open circles).

The number of circuits with fewer than 5 latent fixed-point phenotypes

Similar combinatorial arguments can be made to determine the number of circuits with fewer than 5 latent phenotypes, but the number of attractor landscape isomorphisms and labelings quickly grows unwieldy and the corresponding calculations become increasingly tedious. For example, to calculate the number of circuits with 4 latent phenotypes, one must consider 7 attractor landscape isomorphisms, each with multiple labelings. These calculations are therefore not shown.

Supplementary figures

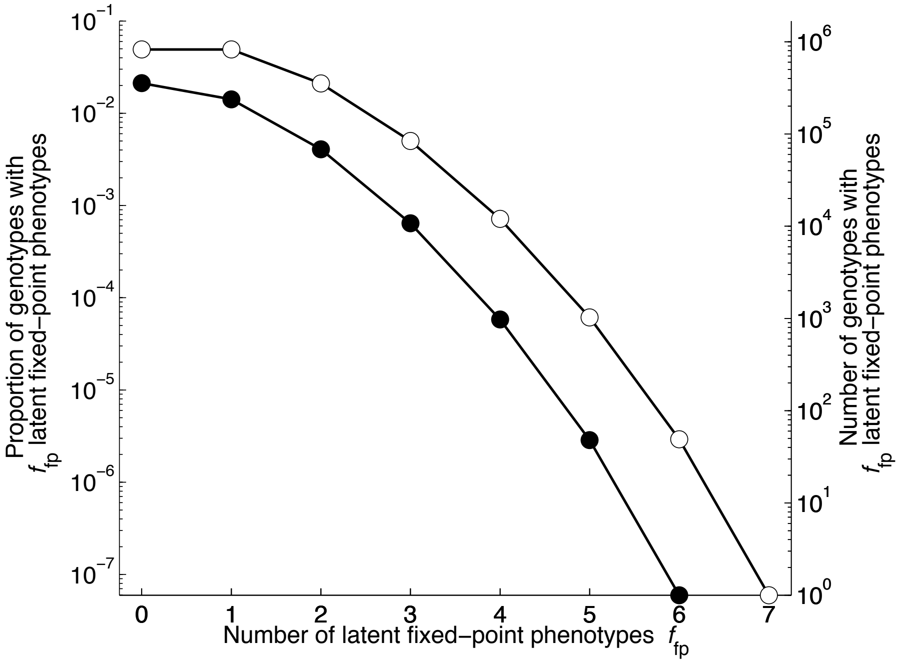

Figure S1 Monofunctional circuits typically have latent fixed-point phenotypes. Each data point depicts the proportion and number of monofunctional circuits with f_{fp} latent fixed-point phenotypes. The white and black circles correspond to identity and transition monofunctions, respectively, as in Fig. 2. The lines are provided as a visual guide. Note the logarithmic scale of the y-axis. Note also that, in contrast to Fig. 2, the number of circuits with $f_{fp} = 0$ latent fixed-point phenotypes differs among identity and transition monofunctions. This is because periodic expression states are not considered to be latent phenotypes here and the number of realizable periodic expression states is greater when only one state is used in the monofunction (i.e., an identity function) than when more than one state is used in the monofunction (i.e., a transition function).

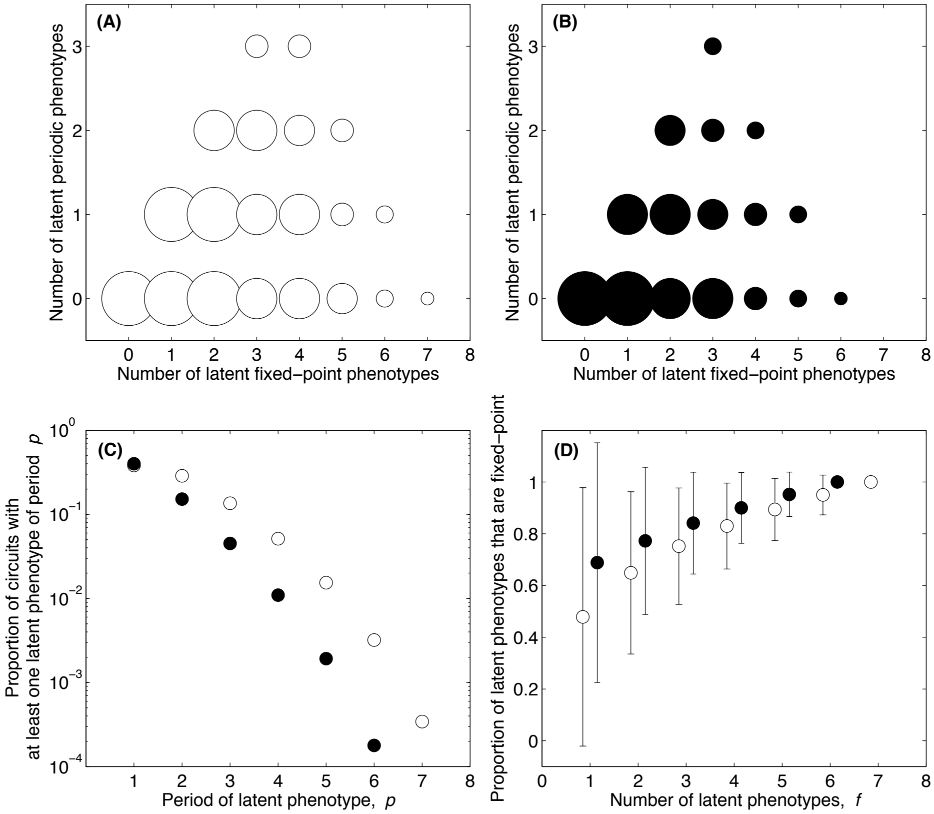

Figure S2 A circuit's latent phenotypes typically consist of a mix of fixed-point and periodic equilibrium expression states. The number of periodic latent phenotypes is shown in relation to the number of latent fixed-point phenotypes for circuits with (A) an identity monofunction ($\langle 0,0,0\rangle \mapsto \langle 0,0,0\rangle$) and (B) a transition monofunction ($\langle 0,0,0\rangle \mapsto \langle 0,0,1\rangle$). Vertex size is proportional to the number of circuits with that combination of periodic and latent fixed-point phenotypes. (C) The proportion of circuits with at least one latent phenotype of period p decreases exponentially with p for both identity (open circles) and transition (filled circles) monofunctions. These data correspond to the same monofunctions shown in (A,B). (D) The proportion of latent fixed-point phenotypes increases with a circuit's number of latent phenotypes f and is higher for transition monofunctions than for identity monofunctions.

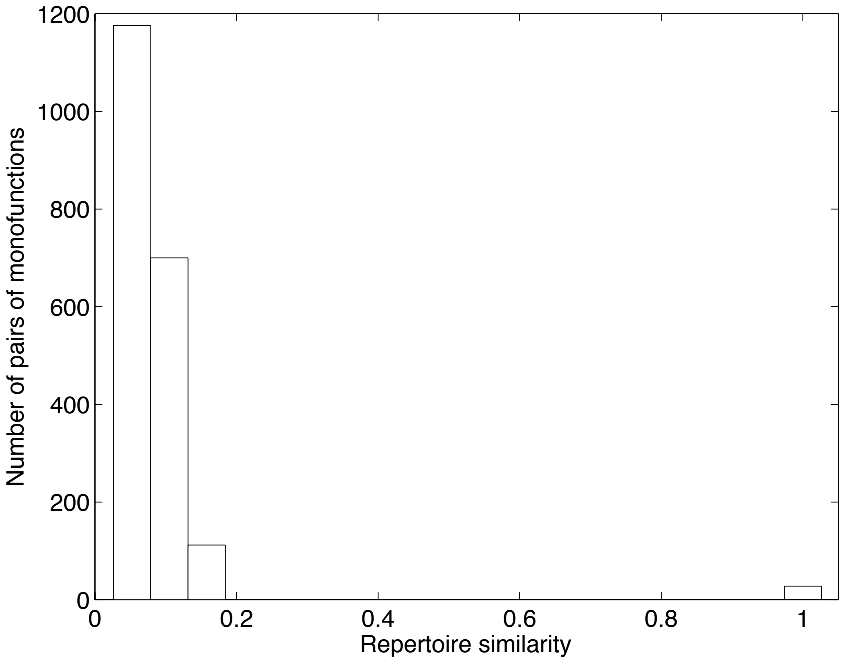

Figure S3 There is little overlap among the latent repertoires of different monofunctions. Data correspond to all 2016 possible pairings of the 64 monofunctions. The height of each bar shows the number of such pairs with a given repertoire similarity, measured as the ratio of the number of latent phenotypes in the intersection of the two repertoires to the number of latent phenotypes in the union of the two repertoires. All pairs of monofunctions with a repertoire similarity of 1 are symmetric (e.g., $\langle 0,0,0\rangle \mapsto \langle 0,0,1\rangle$ and $\langle 0,0,1\rangle \mapsto \langle 0,0,0\rangle$).

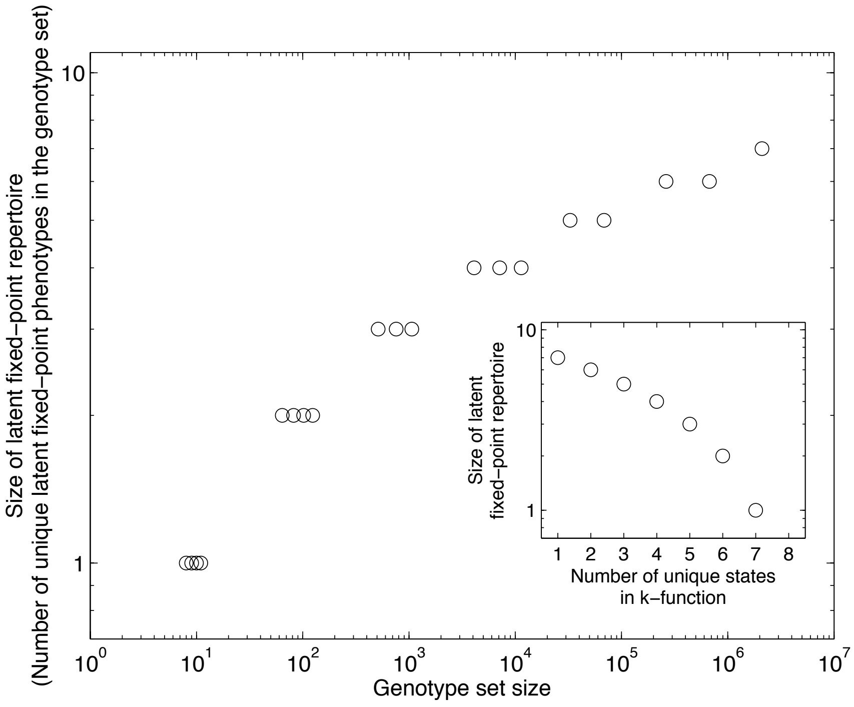

Figure S4 Latent fixed-point repertoire size increases with genotype set size and depends solely upon the number of unique states in the k-function. The size of each k-function's latent fixed-point repertoire is shown in relation to the size of its genotype set. The inset shows the relationship between a k-function's latent fixed-point repertoire size and the number of unique states in the k-function. Note the logarithmic scale of both axes in the main panel and the y-axis of the inset. Note also that the maximum number of latent fixed-point phenotypes is 7, because three-gene circuits have only 8 unique states and at least one of these is part of the circuit's k-function.

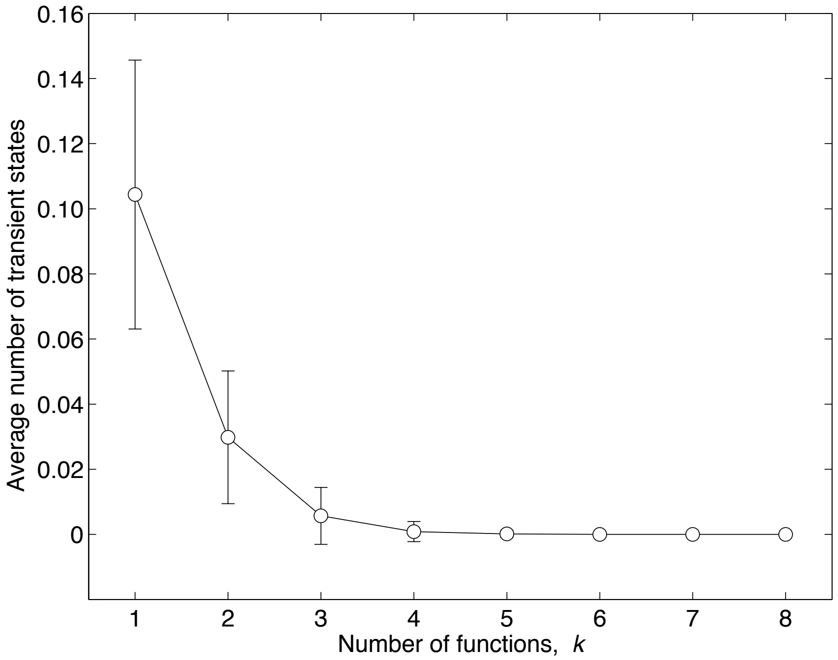

Figure S5 The average number of transient states encountered along a circuit's dynamical trajectory to a latent phenotype decreases with a circuit's number of functions, k. Each data point represents the average number of transient states per latent phenotype per circuit, among all circuits with k functions. Error bars represent a single standard deviation.

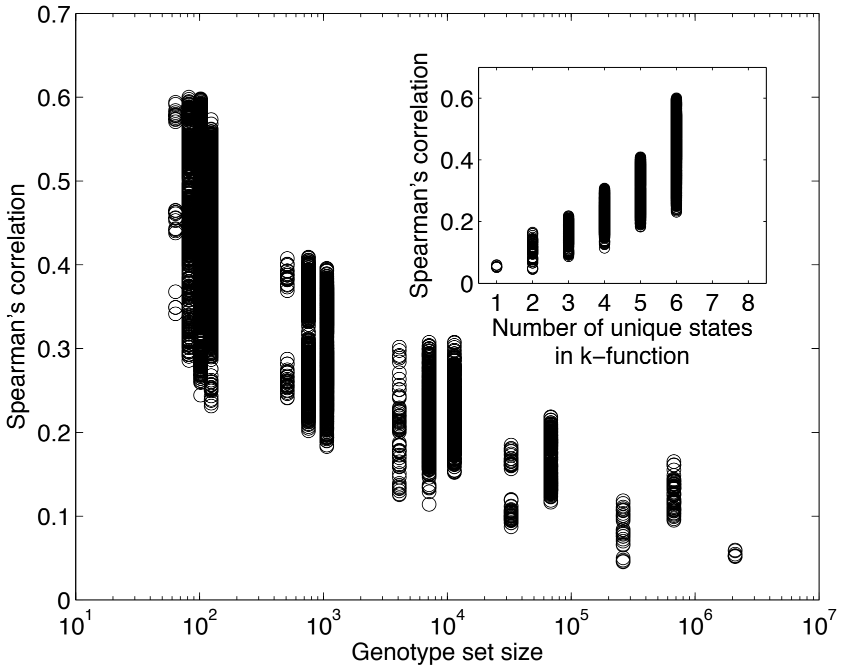

Figure S6 The fraction δ of unique latent phenotypes per pair of genotypes is significantly correlated with the mutational distance between the genotypes, for all k-functions with a latent repertoire size greater than one. Each data point corresponds to 100,000 sampled pairs of genotypes from the dominant genotype network of a single k-function and represents the Spearman's correlation coefficient between the fraction δ of unique latent phenotypes per pair of sampled genotypes and the mutational distance between these genotypes. No correlations are reported for k-functions composed of more than six unique states, because these have a latent repertoire size of one or zero (Fig. 3, inset) and it is therefore not possible to observe any diversity in the latent phenotypes of these circuits. All correlations are statistically significant ($p < 0.001$). Note the logarithmic scale of the x-axis.

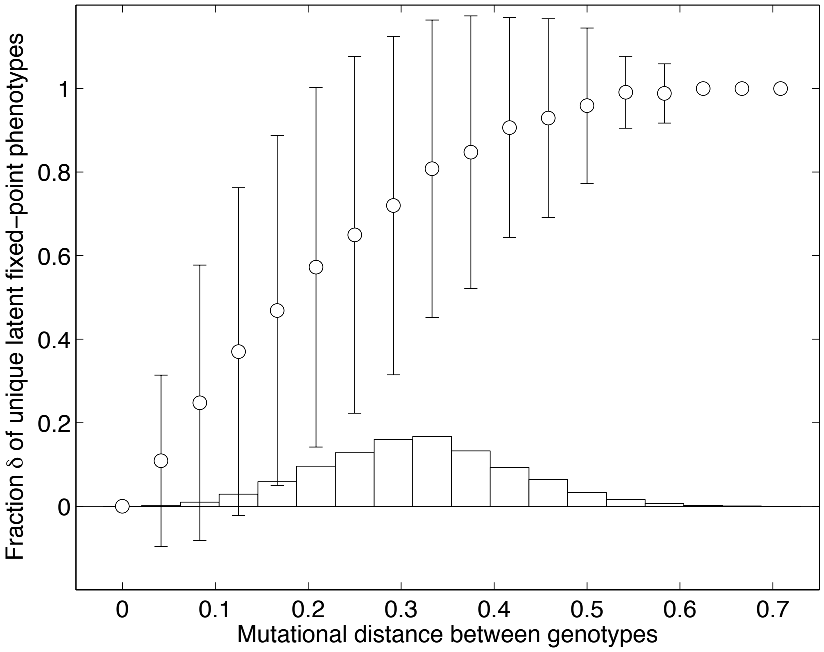

Figure S7 Latent fixed-point phenotypes vary within the dominant genotype network of a k-function. The data shown is based on the same 100,000 sampled pairs of genotypes from the same dominant genotype network shown in Fig. 4. Open circles depict the mean fraction δ of latent fixed-point phenotypes that are unique to one genotype or the other in each pair (see inset and Methods, Eq. 3), shown in relation to the mutational distance between these genotypes (Spearman's $r = 0.41$,$p < 1 \times 10^{-50}$). Error bars correspond to one standard deviation. The histogram shows the distribution of sampled mutational distances between circuits in a pair.

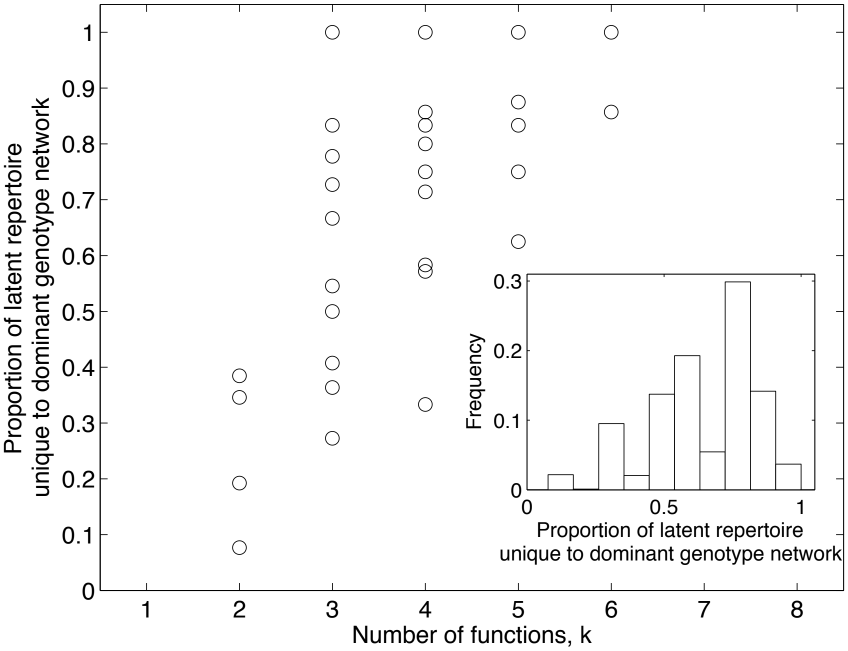

Figure S8 Many latent phenotypes are only found among circuits on the dominant genotype network. Each data point corresponds to a k-function with a fragmented genotype set and depicts the proportion of the k-function's latent repertoire that is exclusive to circuits in the dominant genotype network, shown in relation to the number of functions k (horizontal axis). The inset shows this proportion as a histogram for all 22,160 k-functions with a fragmented genotype set.

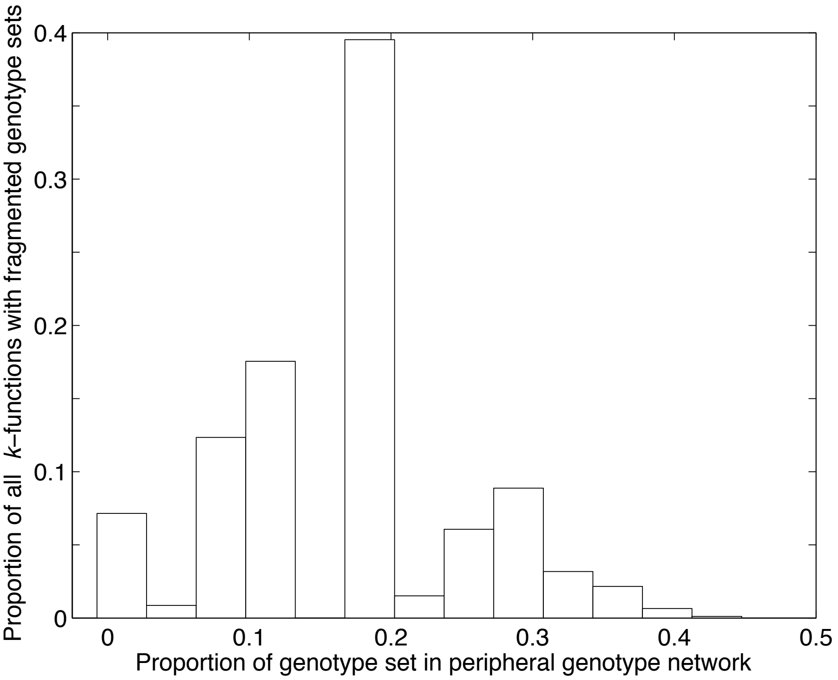

Figure S9 Peripheral genotype networks typically comprise only a small minority of circuits. **Histogram of the proportion of all circuits with a given k-function that belong to a peripheral genotype network. Data corresponds to all 22,160 k-functions with a fragmented genotype set.**

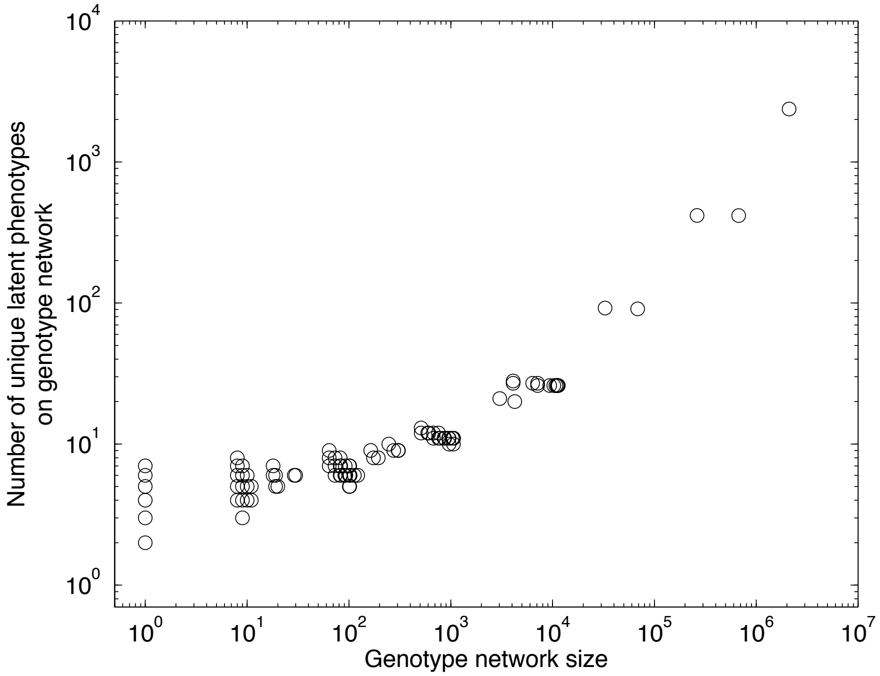

Figure S10 The number of distinct latent phenotypes per genotype network increases with genotype network size. Each data point corresponds to the number of distinct latent phenotypes realized by the circuits on a given genotype network, shown in relation to the size of the genotype network. Data correspond to all $32,399$ k-functions, and thus include fragmented and non-fragmented genotype sets alike. Note the logarithmic scale of both axes.

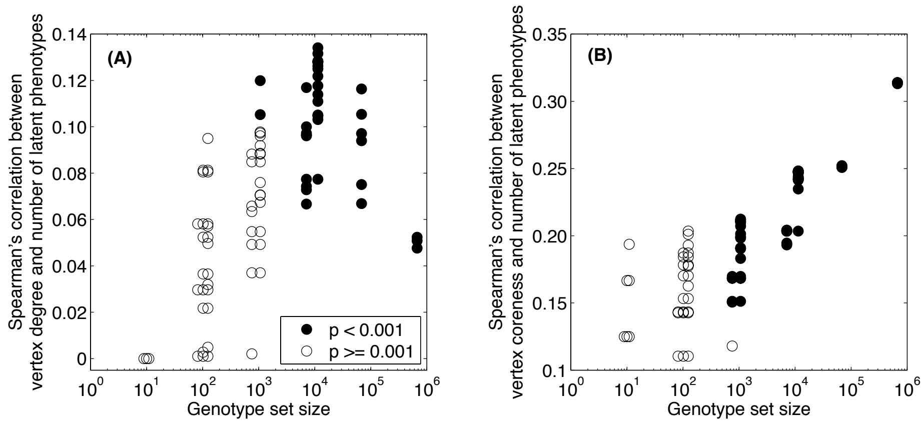

Figure S11 Vertex degree and coreness are statistically associated with the number of latent phenotypes per circuit. Data is shown for all 22,160 k-functions with fragmented genotype sets. (A) The strength of the association between vertex degree and number of latent phenotypes exhibits a non-monotonic relationship with genotype set size. (B) The strength of the association between vertex coreness and the number of latent phenotypes increases with genotype set size. The legend in (A) applies to both panels. Note the logarithmic scale of the x-axes.

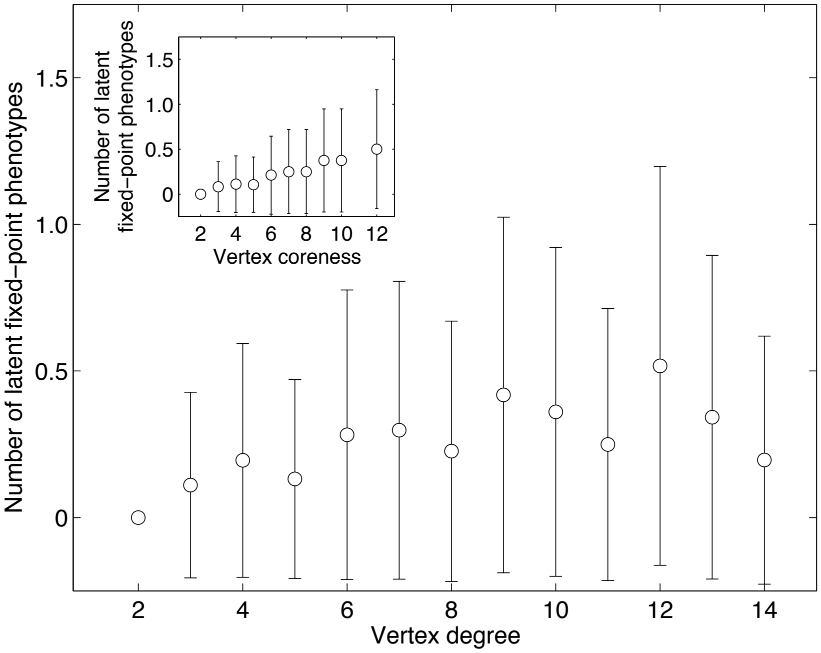

Figure S12 Robust genotypes have many latent fixed-point phenotypes. Data are based on the same bifunction considered in Fig. 5. Open circles depict the mean number of latent fixed-point phenotypes among all genotypes with a given vertex degree (main panel; Spearman's $r = 0.09$,$p = 1.47 \times 10^{-22}$) or coreness (inset; Spearman's $r = 0.19$,$p < 1 \times 10^{-50}$). Error bars correspond to a single standard deviation.

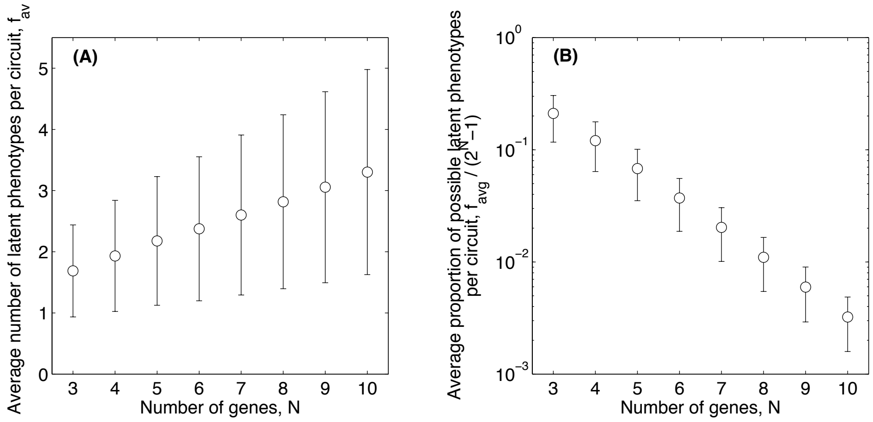

Figure S13 The number, but not proportion, of latent phenotypes per circuit increases with the number of genes. Each data point corresponds to the average of 10,000 sampled genotypes of monofunctional circuits with N genes. Error bars denote a single standard deviation. (A) The average number of latent phenotypes per circuit and (B) the average proportion of possible latent phenotypes per circuit are shown in relation to the number of genes N. Since at least one expression state is used in each monofunction, the maximum number of initial states that may lead to a latent phenotype is $2^N - 1$. This number is therefore used to calculate the proportion of possible latent phenotypes (denominator in y-axis). Note the logarithmic y-axis in (B).
